# ﻿The mitochondrial genome of the bioluminescent fish *Malacosteusniger* Ayres, 1848 (Stomiidae, Actinopterygii) is large and complex, and contains an inverted-repeat structure

**DOI:** 10.3897/zookeys.1157.97921

**Published:** 2023-04-05

**Authors:** Romain Gastineau, Christian Otis, Brian Boyle, Claude Lemieux, Monique Turmel, Jérôme St-Cyr, Marcel Koken

**Affiliations:** 1 Institute of Marine and Environmental Sciences, University of Szczecin, Szczecin, Poland; 2 Plateforme d’Analyse génomique, Institut de Biologie Intégrative et des Systèmes, Université Laval, Québec City, Québec, Canada; 3 Département de biochimie, de microbiologie et de bio-Informatique, Institut de Biologie Intégrative et des Systèmes, Université Laval, Québec, Québec City, Canada; 4 LABOCEA R&D – Centre national de la recherche scientifique’CNRS, 120 Avenue Alexis de Rochon, 29280, Plouzané, France

**Keywords:** Bioluminescence, Deep Sea Dragonfish, inverted-repeat, long-read sequencing, Malacosteinae, mitogenome, Stomiidae

## Abstract

We determined the complete mitogenome sequence of the bioluminescent fish *Malacosteusniger* using long-read sequencing technologies. The 21,263 bp mitogenome features a complex structure with two copies of a 1198-bp inverted-repeat and a region of 2616-bp containing alternating copies of 16 and 26 bp repeat elements. Whole mitogenome phylogenies inferred from both nucleotide and amino-acid datasets place *M.niger* among Melanostomiinae. The need for additional complete mitogenome sequences from the subfamily Malacosteinae is discussed.

## ﻿Introduction

Sunlight is filtered by seawater and only blue/green light with wavelengths between 460 and 490 nm penetrates into the deep sea. Many deep-sea animals with well-adapted eyes can see this very weak light down to about 1500 m (Warrant 2004; de Busserolles et al. 2020). Most of them detect only these blue-green colors, and often produce themselves similar colors by bioluminescence for three main functions: attack, defense/camouflage, and communication.

**Figure 1.**
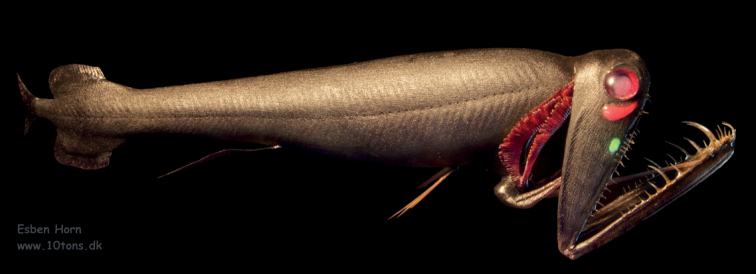
*Malacosteusniger* model made by 10TONS (www.10tons.dk). These most peculiar and unique fishes are frequently damaged by fishing gear and this model allows for the first time to visualize a faithful representation. Courtesy of Esben Horn.

**Figure 2.**
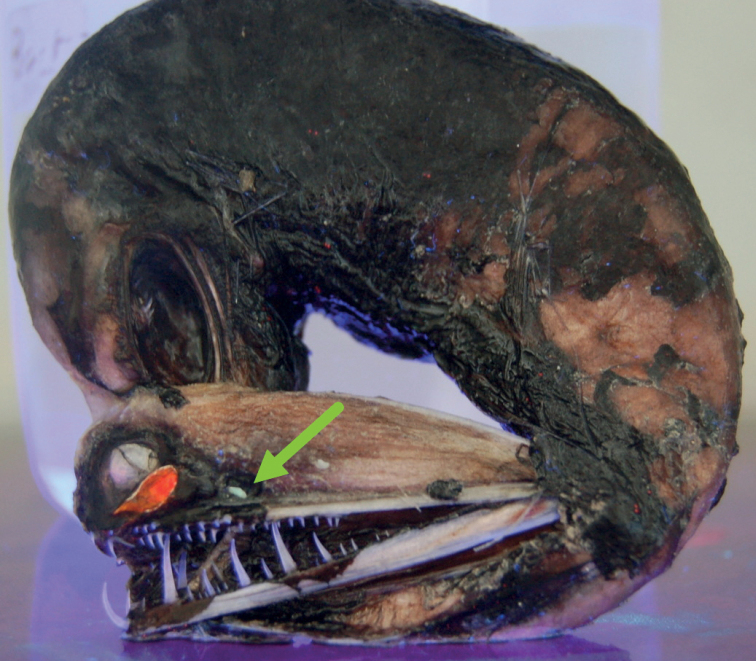
A specimen of *Malacosteusniger* illuminated from below by UV light (360 nm) clearly showing the fluorescence of the suborbital red and the green postorbital eye gland (arrow). The specimen has been damaged during capture.

The peculiar mesopelagic black loose-jaw dragonfish, *Malacosteusniger*Ayres 1848 (Figs 1, 2), represents one of the very few exceptions to this “blue-light rule” (Ayres 1848, 1849; Crossman 1960). This animal lives between 600 and 1000 m and possesses two light-emitting eye glands (Figs 1, 2) (Kenaley 2007, 2008). The suborbital deep-red-emitting photophore is thought to function in intra-specific communication and as a “private torchlight” to detect prey items that are unable to see red light (Mensinger and Case 1988). A bioluminescence reaction in this red eye gland produces blue light that is never emitted, because its energy is transferred to red light-emitting fluorescent proteins via a Bioluminescence Resonance Energy Transfer (BRET) reaction (Campbell and Herring 1987; Herring and Cope 2005). The postorbital eye gland emits blue-green light and is thought to serve intra- and inter-specific exchanges.

Like almost all deep-sea fish, *M.niger* encode only blue opsin photoreceptor genes in order to detect the down welling as well as the bioluminescent light omnipresent in the environment. However, *M.niger* adapted its eyes by linking antenna-pigments (a red-absorbing bacterio-chlorophyll c) to its blue opsin proteins (Douglas et al. 1998a), in order to detect its own red-light emission. Chlorophyll use by vertebrates is very rare and *M.niger* shares this capacity thus far only with a salamander (Isayama et al. 2006). How the fish obtains or produces this chlorophyll is also still an enigma, but it is hypothesized to be retrieved through the food-chain by consuming small copepods which have eaten bacterio-chlorophyll c – containing bacteria (Douglas et al. 1998b, 1999, 2000; Schwab and Marshall 2004). Finally, *M.niger* is the only known vertebrate lacking a hyoid membrane (the membrane that closes the space between the lower jaw), a feature which comes in addition to the unique occipito-vertebral gap observed in several Stomiidae (Schnell et al. 2010; Kenaley 2012; Schnell and Johnson 2017 a, b). For a long time, this 15–20 cm long fish was thought to be a top-predator that mainly eats “big fish”. However, stomach content analysis showed that the animal prefers smaller prey, including the 3–5 mm calanoid copepod *Chirundinastreetsi* Giesbrecht, 1895 (Sutton 2005; Drazen and Sutton 2017). This raises the question about how this animal retains its meal in a seemingly open mouth.

We initiated this study by analyzing the mitogenome of *M.niger* with short read sequencing technologies. However, all our efforts to assemble the complete *M.niger* mitogenome proved unsuccessful. The mitogenome always came out as two distinct contigs of similar coverage and all attempts to join them were fruitless whatever parameters were used for assembly. It quickly appeared that repeated sequences were at the base of this problem and that long-read sequencing technologies were clearly needed to resolve the complex mitochondrial genome. The results presented here show yet another peculiarity of *M.niger* with the discovery of a large and complex mitochondrial genome harboring inverted-repeat-like structures.

## ﻿Materials and methods

### ﻿Biological material and DNA extraction

The *Malacosteusniger* specimen used in this study was caught during the Bear Seamount cruise DE200611 (station 012, 18/6/2006). Pieces of the caudal fin and muscle were sent to the “Plateforme d’Analyses Génomiques” of the “Institut de Biologie Intégrative et des Systèmes” of the Université Laval (Québec, Canada) for DNA library preparation and sequencing. For DNA extraction, 300 mg of muscle were crushed in liquid nitrogen and digested at 65 °C for 30 min in 1.0 ml lysis buffer containing 50 mM Tris-HCl pH 8.0, 200 mM NaCl, 20 mM EDTA, 2.0% SDS and 20 mg/ml proteinase K. An equal volume of CTAB buffer containing 50 mM Tris-HCl pH 8.0, 1.4 M NaCl, 20 mM EDTA, 2.0% CTAB, 1.0% PVP 40,000 was added to the lysate and incubation was pursued for an additional 30 min at 65 °C. This mixture was extracted with phenol: chloroform: isoamylalcohol (25:24:1), and following centrifugation, 5 µl of RNase A (100 mg/ml) was added to the aqueous phase and incubated at room temperature for 20 min. This mixture was then extracted twice with an equal volume of chloroform: isoamylalcohol (24:1) and DNA was precipitated with two volumes of EtOH, dried and dissolved in 100 µl of TE buffer (10 mM Tris-HCl pH 8.0, 0.1 mM EDTA).

### ﻿Short reads sequencing

The library preparation protocol for short reads sequencing was as follows. Genomic DNA (500 ng in 55 ul TE buffer) was mechanically fragmented for 40 s using a Covaris M220 (Covaris, Woburn MA, USA) with default settings. Fragmented DNA was transferred to PCR tubes and library synthesis was performed using a NEB Next Ultra II kit (New England Biolabs) according to the manufacturer’s instructions. To barcode the samples, TruSeq HT adapters (Illumina, SanDiego, CA, USA) were used. The library was sequenced on the Illumina MiSeq platform (300-bp paired-end reads). Of the 15 335 342 raw paired-end reads obtained, 11 121 576 remained after elimination of low-quality reads.

### ﻿Long reads sequencing

For long-read sequencing, DNA was quantified using a Qubit fluorometer (ThermoFisher) and quality checked on a Femto Pulse System with a genomic DNA 165-kb kit (Agilent, Santa Clara, CA, USA). A DNA aliquot of 8 µg was fragmented with a Covaris g-tube (Covaris Woburn, MA, USA) and small fragments were removed using Short Read Eliminator XS (Circulomics/PacBio, Menlo Park, CA, USA). The library for Oxford Nanopore MinIon sequencing was prepared using 1.69 µg of DNA and the LSK-109 ligation sequencing kit (Oxford Nanopore, Littlemore, UK), following repair and end-polishing of the sheared DNA using the NEBNext Companion Module for Oxford Nanopore Technologies ligation kit (New England Biolabs, Ipswich, MA, USA). Finally, 0.595 µg of library were loaded on a R9.4.1 MinION flow cell and sequencing was performed on a GridIon benchtop platform (Oxford Nanopore).

### ﻿Assembly and annotation

All bioinformatics analyses were performed on the THOT superdome flex server at “Université Laval”. MiSeq reads were first processed with AfterQC (Chen et al. 2017) to remove adapters and low-quality reads. They were assembled using SPAdes v.3.15.5 (Bankevich et al. 2012), with a k-mer parameter of 125. Data mining in the pool of contigs was done with blastn command line (Camacho et al. 2009). Boundaries of the contigs were extended using the addSolexaReads.pl script of Consed (Gordon and Green 2013). Basic statistics of the Nanopore reads were obtained with NanoStat (De Coster et al. 2018). Nanopore reads were first filtered using Filtlong v.0.2.1 (https://github.com/rrwick/Filtlong), with the two contigs obtained from short reads as a reference. The filtered reads were assembled using Canu v.2.2 (Koren et al. 2017) with a genome size parameter of 0.05M. The contig obtained from Canu was polished with Pilon v.1.24 (Walker et al. 2014) using the MiSeq paired-end reads. Polishing with Pilon was stopped after four iterations, the last one leading to a single base correction.

Annotation was performed with the help of MITOS (Bernt et al. 2013) and manually curated. tRNA genes were identified using Arwen v.1.2 (Laslett and Canbäck 2008). The exact boundaries of the inverted-repeat-like structure were found using the LAST aligner (Kiełbasa et al. 2011). The map of the organellar genome was obtained with OGDRAW (Lohse et al. 2013). The sequences corresponding to the inverted-repeat like structure and the simple repeat portion were displayed with WebLogo v.3 (Crooks et al. 2004).

### ﻿Maximum likelihood phylogeny

Protein-coding genes and the corresponding amino-acid sequences were extracted from the mitochondrial genomes of 15 taxa of Stomiiformes, including *M.niger*. *Xiphiasgladius* Linnaeus, 1758 was used as an outgroup. Nucleotide and inferred amino-acid sequences of all conserved mitochondrial genes (*ATP6*, *ATP8*, *cox1*, *cox2*, *cox3*, *cytB*, *ND1*, *ND2*, *ND3*, *ND4*, *ND4L*, *ND5*, *ND6*) were first concatenated for each species/dataset and then aligned using MAFFT 7 (Katoh and Standley 2013) with the “-auto” option. Poorly aligned regions were filtered out with trimAl (Capella-Gutiérrez et al. 2009) using the “-automated1” option, and the trimmed version of each data set was used to determine the best model of nucleotide and amino-acid evolution with ModelTest-NG (Darriba et al. 2020). Maximum likelihood phylogenies were obtained using IQ-TREE v.2.2.0 (Minh et al. 2020), with 10 000 bootstrap replications in both cases.

### ﻿Data resources

The mitochondrial genome has been submitted to GenBank with accession number OP326280. The raw fasta file, the annotated gbk file and a fastq file containing the longest Oxford Nanopore read supporting the assembly can be found on Zenodo following this link: https://doi.org/10.5281/zenodo.7330521.

## ﻿Results

### ﻿*Malacosteusniger* mitogenome assembly using short sequencing reads

The size and sequence coverage of the mitogenome contigs obtained after SPAdes assembly of short sequencing reads are indicated in Table 1. Two contigs containing protein-coding and ribosomal RNA genes were retrieved by blastn analyzes using mitogenome data from other Stomiiformes as queries, while the two other contigs were found by extending the end sequences of the former contigs using Consed. The 1198 bp contig displayed twice the coverage of other contigs and could be placed in an inverted-repeat position at both ends of the 12 311 bp contig. One copy of the inverted repeat also proved to be linked to the 4467 bp contig. The 750 bp contig shared short sequences with the inverted repeat but this overlap remained ambiguous.

**Table 1.** Sizes, coverage and gene contents of the *M.niger* mitogenome contigs obtained after assembly of short sequencing reads.

Size of the contig (bp)	12 311	4467	1198	750
Coverage	162.46X	156.70X	270.75X	121.78X
Gene content	*ND2*, *cox1*, *cox2*, *ATP8*, *ATP6*, *cox3*, *ND3*, *ND4L*, *ND4*, *ND5*, *ND6*, cob, 18 tRNA genes	*ND1*, *rrnS*, *rrnL*, 3 tRNA genes	None	None

### ﻿Assembly of the complete *M.niger* mitogenome using long sequencing reads

Oxford Nanopore sequencing was undertaken to confirm and resolve the contig overlaps that were identified using the short read approach. Table 2 shows the statistics of the Nanopore reads obtained before and after Filtlong filtering on the reference 12 311 and 4467 bp contigs. Canu assembly of the filtered reads returned a single 24 086 bp contig with overlapping end sequences. A 21 263 bp contig representing the complete mitogenome remained after trimming this overlap. It is worth noting that a single Nanopore read of 20 972 bp covered nearly the complete genome (Table 2).

**Table 2.** Basic statistics of the Nanopore reads before and after Filtlong filtering.

	Nanopore reads (before filtering)	Nanopore reads (after filtering)
Mean read length (bp)	3854	4093
Mean read quality	13.2	14.4
Median read length (bp)	1754	3304.0
Median read quality	13.2	14.4
Number of reads	212 934	153
Read length N50 (bp)	8515	6327
Total bases (bp)	820 590 847	626 269
Top 5 longest reads (bp)	78 432, 77 491, 63021, 58 837, 58 554	20 972, 17 597, 14 526, 14 300, 12 821

### ﻿Structure and gene content of the *M.niger* mitogenome

**Figure 3.**
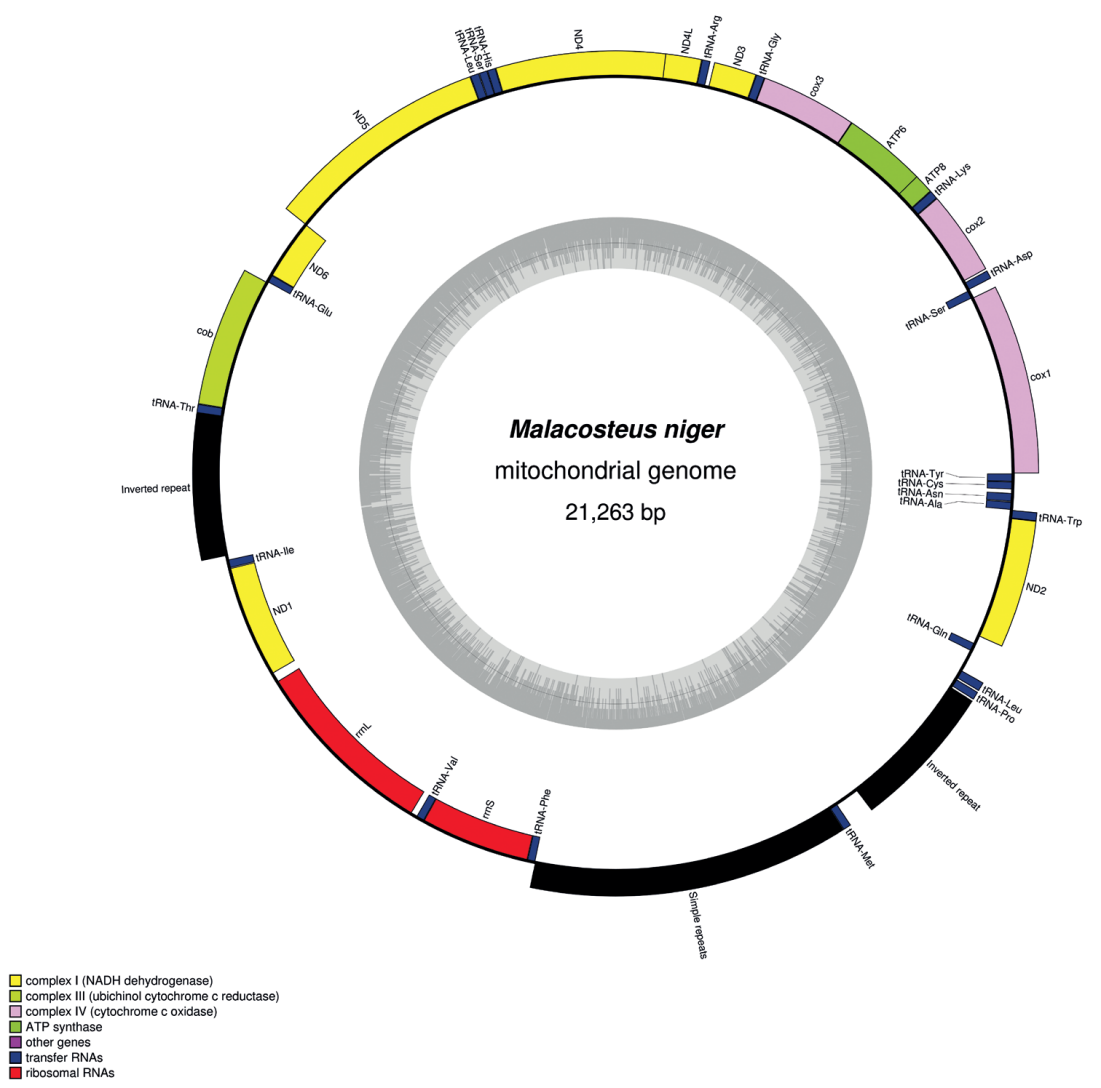
Map of the mitochondrial genome of *Malacosteusniger*. The type of genes are indicated in the legend. The additional black boxes correspond to the two inverted-repeat structures and the long single-repeats fragment.

**Figure 4.**
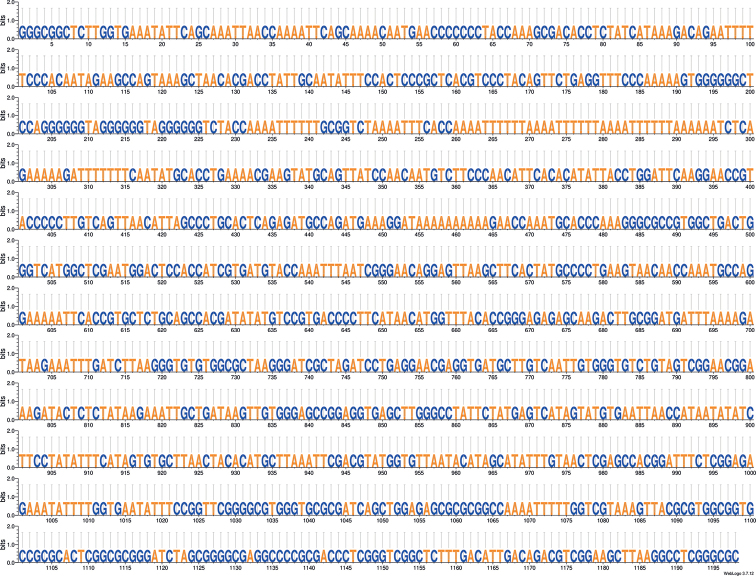
Sequence of the 1198 bp inverted-repeat structure found in the mitochondrial genome of *Malacosteusniger*.

The 21 263 bp mitogenome of *M.niger* (GenBank: OP326280) contains 46% G+C and encodes 13 proteins, 22 tRNAs and 2 rRNAs (Fig. 3). It features a 1198 bp inverted-repeat structure with 44% G+C (Fig. 4); one copy of this repeat is located between the genes encoding tRNA-Thr and tRNA-Ile, while the second copy is located between the genes encoding *tRNA-Met* and *tRNA-Pro*. Another region rich in repeated elements is found between the genes encoding *tRNA-Phe* and *tRNA-Met*. This region of 2616 bp contains 36% G+C and features repeat elements of 41 and 26 bp (Table 3): The 41-bp and 26-bp elements are repeated 16 and 26 times, respectively, at positions that alternate between the two elements. There is an overlap between *ATP6* and *ATP8* and also between *ND4L* and *ND4*. Finally, it should be noted that the *cox2*, *ND4* and *cob* genes feature stop codons that are carried by flanking sequences of tRNA genes. Genes are coded on both strands. Our long-read supported assembly proves that *ND1*, *rrnL* and *rrnS* are on the opposite strand in contrast to most other genes.

**Table 3.** Sequences of the repeat elements identified in the region of the *M.niger* mitogenome located between the *tRNA-Met* and *tRNA-Phe* genes.

Sequence of the repeated motif	Number of occurrences
(5’-CATATATCAATATCGACATATGTCAATATTGACATATATCA-3’)	16
(5’-GTCAATACAAACGCATGTGTTTTTAT-3’)	26

### ﻿Phylogenic position of *M.niger* among the Stomiiformes

**Figure 5.**
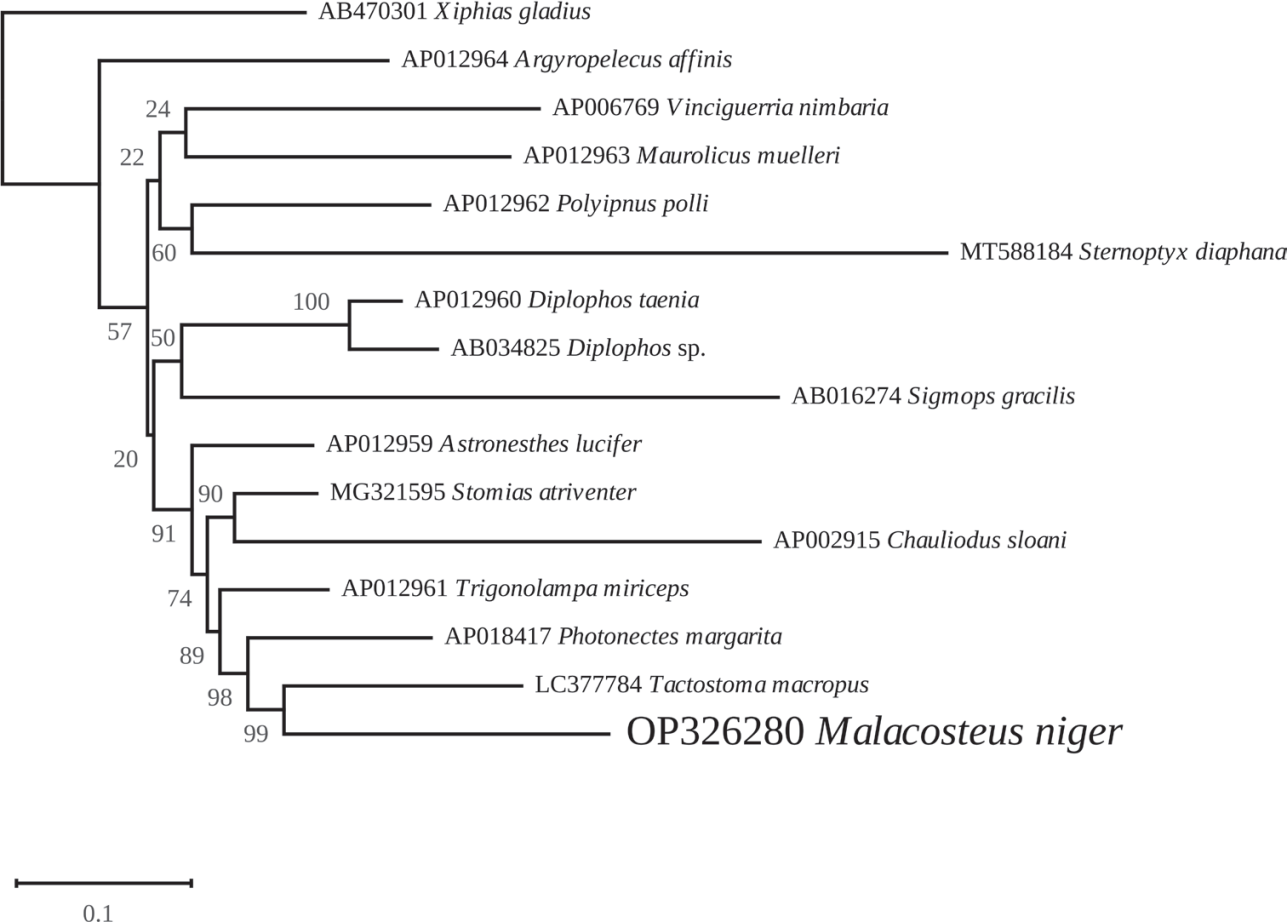
Maximum likelihood phylogenetic tree obtained from concatenated amino-acid sequences of the mitochondrial proteins of *Malacosteusniger* and other Stomiiformes, with a phylogenetically very distant carangiform *Xiphiasgladius* as an outgroup.

**Figure 6.**
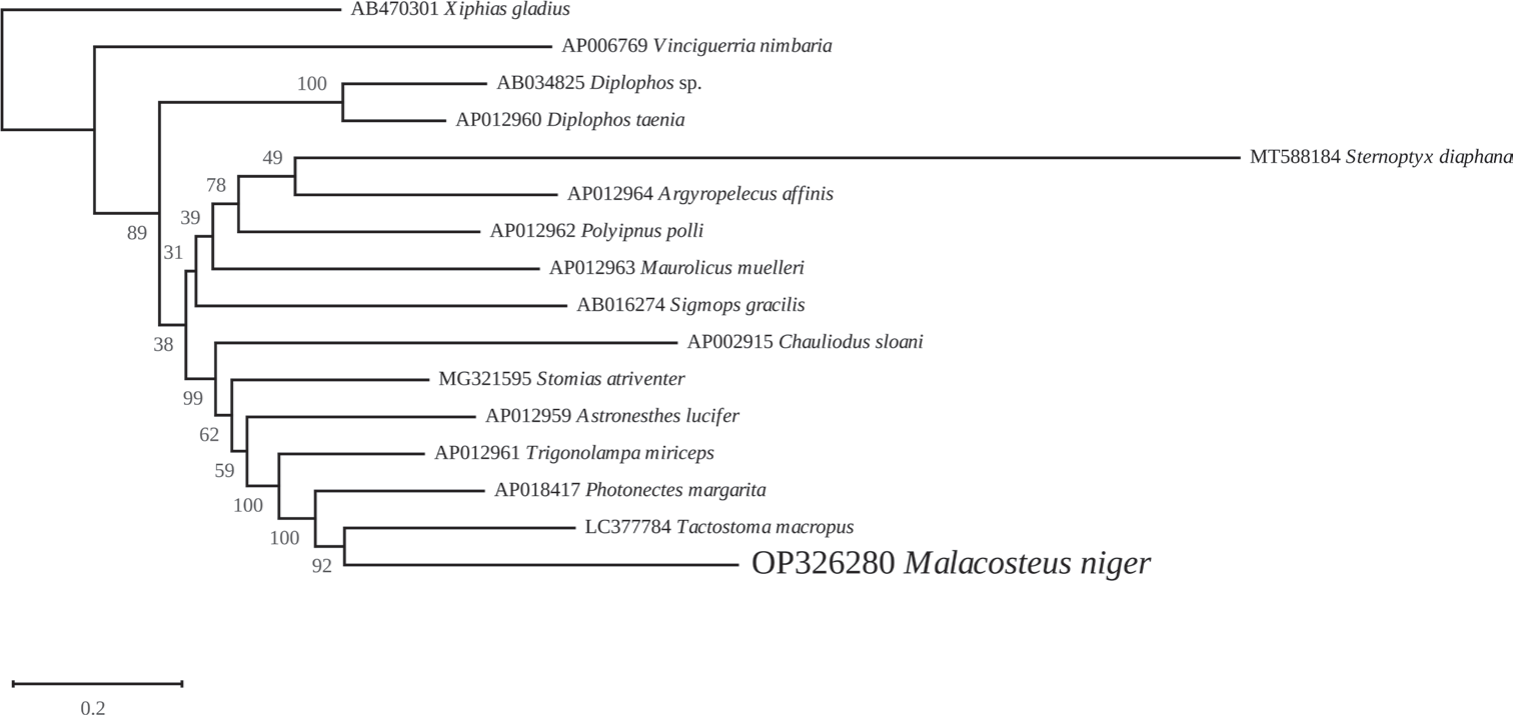
Maximum likelihood phylogenetic tree obtained from concatenated nucleotide sequences of the mitochondrial proteins of *Malacosteusniger* and other Stomiiformes, with a phylogenetically very distant carangiform *Xiphiasgladius* as an outgroup.

Phylogenetic analyses of the 11 411 nucleotides and 3795 amino-acid datasets were conducted using the GTR+I+G and mtMAM+I+G+F evolutionary models, respectively. Separation between the Stomiidae and Gonostomatidae was weakly supported in trees inferred from both datasets, but several nodes within the Stomiidae clade proved to be more robust, especially in the amino acid inferred phylogeny. Both the nucleotide and amino-acid trees revealed that the Malacosteinae*M.niger* is sister to the Melanostomiinae*Tactostomamacropus* Bolin, 1939 (LC377784), forming a clade with the Melanostomiinae*Photonectesmargarita* (Goode & Bean, 1896) (AP018417) and *Trigonolampamiriceps* Regan & Trewavas, 1930 (AP012961). This group of taxa is sister to a clade containing the Stomiinae*Stomiasatriventer* Garman, 1899 (MG321595) and the Chauliodontinae*Chauliodussloani* Bloch & Schneider, 1801 (AP002915) in the amino-acid phylogeny (Fig. 5), and to the Astronesthinae*Astronestheslucifer* Gilbert, 1905 (AP012959) in the nucleotide phylogeny (Fig. 6).

## ﻿Discussion

With 21 263 bp, the mitogenome of *M.niger* is among the largest described so far among the Actinopterygii. Recent studies have unveiled very large mitochondrial genomes among metazoan invertebrates, with a current record of 93 065 bp being held by the parasitic cnidarian *Polypodiumhydriforme* Ussov, 1885 (MN794187) (Novosolov et al. 2022), closely followed by the 67 195 bp of the zebra mussel *Dreissenapolymorpha* (Pallas, 1771) (CM035931) (McCartney et al. 2022). Among Chordata, the largest known mitogenomes belong to the amphibian *Brevicepsadspersus* Peters, 1882 (AB777216) and the Actinopterygii*Drepanepunctata* (Linnaeus, 1758) (KM273123), which reach 28 757 and 23 152 bp, respectively.

Inverted-repeat structures are a common feature among plastomes (Turmel and Lemieux 2018), with a few exceptions such as the microalgae Pelagophyceae (Ong et al. 2010) and some species of the Dictyochophyceae (Han et al. 2019). However, the presence of such structures in mitogenomes is unusual. To our knowledge, mitogenome inverted repeats have been reported so far only among Stramenopiles and Basidiomycetes (Nieuwenhuis et al. 2019), and a few Chlorophyceae (Robbens et al. 2007; Worden et al. 2009; Pombert et al. 2013; Satjarak et al. 2017; Turmel et al. 2020). Inverted repeats have been identified in some metazoan mitogenomes but their sizes rarely exceed 30 bp (Čechová et al. 2018).

Unveiling unusual features in mitogenomes often faces technical limitations, such as those described in the current study that resulted from the use of short sequencing reads. Discovery of metazoan mitogenomes with anomalous characteristics will certainly become more common with increased use of long-read sequencing. In recent studies, long-read sequencing has been decisive in resolving the complex control regions of mitogenomes from Gastropoda (De Vivo et al. 2022) and trematodes (Kinkar et al. 2021), and to assess the presence of two mitochondrial chromosomes in the isopod *Isocladusarmatus* H. Milne Edwards, 1840 (Pearman et al. 2022) and the Tuatara lizard, *Sphenodonpunctatus* Gray, 1842 (Macey et al. 2021).

Additional mitogenome sequences from the Malacosteinae are clearly needed to resolve the phylogenetic position of these bioluminescent fishes. *Malacosteusniger* is the only representative of the Malacosteinae that has been sampled so far among the three genera described in this subfamily. It will be particularly important to analyze the mitogenomes from the two remaining genera (*Aristostomias* Zugmayer, 1913 and *Photostomias* Collett, 1889) that harbor a total of 14 valid species. These studies are expected to shed light not only on the phylogenetic positions of these bioluminescent fishes but also on the putative presence, origin and evolution of the inverted-repeat structure among the mitogenomes of Malacosteinae.
